# Lower Urinary Tract Symptoms in Subjects with Subclinical Cerebral White Matter Lesions

**DOI:** 10.1155/2018/1582092

**Published:** 2018-07-30

**Authors:** Chi-Hang Yee, Ching Leung, Yuki Yu-Ting Wong, Sylvia Lee, Jenny Li, Pauline Kwan, Winnie Chiu-Wing Chu, Vincent Chung-Tong Mok, Chi-Fai Ng

**Affiliations:** ^1^Prince of Wales Hospital, 30-32 Ngan Shing Street, Shatin, New Territories, Hong Kong; ^2^Department of Surgery, SH Ho Urology Centre, The Chinese University of Hong Kong, Hong Kong; ^3^The Chinese University of Hong Kong, Hong Kong; ^4^Department of Medicine and Therapeutics, The Chinese University of Hong Kong, Hong Kong; ^5^Department of Imaging and Interventional Radiology, The Chinese University of Hong Kong, Hong Kong

## Abstract

**Aim:**

We assessed the impact of cerebral white matter lesions (WMLs) on lower urinary tract symptoms in subjects with normal neurological and cognitive function.

**Methods:**

A cohort of community-dwelling subjects aged ≥65 years were recruited to undergo MRI brain assessment. WMLs were graded using the Fazekas scale from 0 to 3. A separate telephone interview was carried out to assess the urinary symptoms in these subjects using the questionnaire Overactive Bladder-Validated 8-Question Awareness Tool (OAB-V8).

**Results:**

800 community-dwelling elderly subjects were recruited to undergo MRI brain. In this cohort, 431 subjects responded to the telephone interview concerning their urinary symptoms. Among the respondents, 21.1% did not exhibit any WML on their MRI brain. Most of the subjects (52.6%) exhibited grade 1 WML. On logistic regression, age was found to be positively correlated with the Fazekas score (correlation coefficient 0.203, *p* ≤ 0.01). Using a cutoff of 8 on OAB-V8, 22% of the respondents experienced OAB. Presence of WML, hypertension, or diabetes mellitus was not found to be correlated with storage urinary symptoms or OAB-V8 total score. Multiple logistic regression analysis did not show the presence of WML to be associated with the diagnosis of OAB (adjusted OR 1.13, 95% CI 0.65–1.96, *p*=0.659).

**Conclusions:**

WML is associated with age and is common in the elderly population. Mild WML is subclinical, with no obvious neurological and urinary symptoms. Our cohort did not demonstrate a relationship between WML and lower urinary tract symptoms.

## 1. Introduction

Overactive bladder (OAB) syndrome is defined as “urgency, with or without urge incontinence, usually with frequency and nocturia” [1], and its incidence is high in population above 40 years [2, 3]. While aging is an underlying factor for OAB syndrome, there are multiple ways which can contribute to the happening of these lower urinary tract symptoms. These include altered lower urinary tract sensory input [4], overactive detrusor contractions resulting from increased excitability and spread of contraction within the muscle [5], and an abnormality in the central nervous system that causes a generalized, nerve-mediated, excitation of the detrusor muscle [6]. On aging, there is an increased risk of atherosclerosis [7]. It brings bladder ischaemia. Subsequent reperfusion injures nerves, resulting in smooth muscle damage and impaired contractility. Overactive bladder syndrome would be a consequence [8].

Atherosclerosis is also a systemic condition that can affect cerebral arteries supplying the brain. Cerebral white matter lesion (WML) on magnetic resonance imaging (MRI) is a common finding in the elderly, signifying chronic bilateral ischaemic brain injury [9]. WML progression has been associated with cognitive decline and dementia, motor dysfunction, and depression. Longitudinal studies consistently reported a relationship between WML progression and decline in cognitive performance [10, 11]. Furthermore, WML in the context of cognitive and motor impairment is reported to be associated with urinary incontinence [12]. These dysfunctions were grouped as geriatric syndromes of vascular dementia, vascular parkinsonism, and vascular incontinence. However, the impact of total subclinical WML on the urinary system, that is, WML without any cognitive and motor symptoms, is seldom studied. We report a prospective study on urinary symptoms in a group of otherwise healthy community-dwelling elders with WML, in the absence of any previously documented stroke and dementia.

## 2. Materials and Methods

A cohort of subjects for brain MRI were recruited by means of recruitment notices placed in housing estates and community centers in Hong Kong. In order to avoid undersampling of the subjects of the more advanced age, stratified sampling was adopted in order to have around 33% of subjects in each of the following age strata: 65–69, 70–74, and ≥75 years. Inclusion criteria were as follows: (1) age ≥65 years, (2) community-dwelling elderly, and (3) sufficient language competency for cognitive testing. Exclusion criteria were as follows: (1) history of clinical stroke or transient ischaemic attack and (2) dementia determined by the published cutoff values of the Chinese Mini-Mental State Examination or medical history [13].

Recruited subjects would undergo mini-mental state examination after data on demographics and basic medical health background had been collected. Brain MRI was then performed for all eligible subjects with a 3T scanner (Achieva X-series; Medical Systems, Best, Netherlands). To comply with the VCI harmonization standard [14], 3D/T1- and proton-density/T2 and fluid-attenuated inversion recovery (FLAIR) sequences were obtained for WML measurement, which was determined as ill-defined hyperintensities ≥5 mm on FLAIR but isointense with normal brain parenchyma on T1-weighted images. Using the Fazekas scale, deep white matter hyperintense signals were rated as 0 = absence, 1 = punctate foci, 2 = beginning confluence of foci, or 3 = large confluent areas [15].

A separate telephone interview was carried out to assess the urinary symptoms in these subjects with MRI brain performed. The questionnaire Overactive Bladder-Validated 8-Question Awareness Tool (OAB-V8) was used to assess the urinary symptoms of the subjects. The OAB-V8 is a self-administered tool directed toward detecting patients suffering from OAB [16]. It contains the original 8 items from the OAB-q symptom bother scale with modified instructions. To assess a subject's urinary symptoms, the OAB-V8 asks how bothered one is by the four hallmark symptoms of OAB: urinary frequency, urgency, nocturia, and urge incontinence. Items are measured on a 6-point Likert scale (0 =  not at all to 5 =  a very great deal), plus a dichotomous question about the patient's gender, which would add 2 points if the subject is male. The total score ranges from 0 to 42 points, with a score above 8 points reflecting that the patient may have OAB.

Descriptive statistics were used to characterize the demographic data. The generalized linear model of binomial regression with a robust estimator was used to evaluate whether there were any predictors for urinary symptoms of urgency and frequency, as well as OAB-V8 total score. Multiple logistic regression analysis to investigate the correlation between OAB and different potential predictive factors was done, with *p* < 0.05 being considered as statistically significant. SPSS software version 22.0 (SPSS Inc, Chicago, IL, USA) was used for calculations.

## 3. Results

Between September 2011 and August 2014, 800 community-dwelling elderly subjects were recruited to undergo MRI brain. In this cohort, 431 subjects responded to the telephone interview concerning their urinary symptoms, with a response rate of 53.9% (Table 1). The mean age of the respondents was 71 ± 5.1 years. Only 9 patients were on anticholinergics for their OAB symptoms. None was on beta-3 agonist. Among the 431 subjects, 91 (21.1%) of them did not exhibit any WML on their MRI brain. Most of the subjects exhibited a mild degree of WML, yielding grade 1 on the Fazekas scale. On logistic regression, age was found to be positively correlated with the Fazekas score (correlation coefficient 0.203, *p* ≤ 0.01).

In our cohort of respondents, most of the subjects experienced no or very mild urinary symptoms of OAB (Table 2). When we broke down the questions of OAB-V8 into different domains, 67.3% of the respondents did not experience any urgency and 26.5% experienced mild urgency symptoms; 74.7% did not experience any frequency and 13.7% experienced mild frequency symptoms; 49.4% did not experience any nocturia and 31.8% experienced mild nocturia symptoms; and 68.9% did not experience any urge incontinence and 26.7% experienced mild urge incontinence.

Binomial regression with a robust estimator was used to evaluate whether there were any predictors for urinary symptoms of urgency and frequency, as well as OAB-V8 total score (Table 3). Age was found to be statistically significantly correlated with the frequency symptom. Presence of WML, hypertension, or diabetes mellitus was not found to be correlated with storage urinary symptoms or OAB-V8 total score. Using the OAB-V8 total score of >8 as a cutoff point for the diagnosis of OAB, multiple logistic regression analysis did not show the presence of WML to be associated with the diagnosis of OAB (adjusted OR 1.13, 95% CI 0.65–1.96, *p*=0.659) (Table 4).

## 4. Discussion

The first pathological description of WML in the brain of elderly dates back to Durand-Fardel in 1854 [17]. In 1987, Hachinski introduced the descriptive term “leukoaraiosis” to define WML without specifying the pathology or aetiology [18]. The prevalence of WML increases with age. The most accepted opinion on the aetiology of WML is the consequence of ischaemia, which is representing a vascular process linked with cerebral small vessel changes. These changes may also lead to a damaged blood-brain barrier, resulting in a chronic leakage of fluid and macromolecules in the white matter [19].

WMLs are frequent findings on brain imaging. Zimmerman et al. reviewed 365 consecutive MRIs with age ranging from 6 to 84 years [20]. WMLs were found in 93.5% of the patients regardless of the diagnosis. Eighty-seven symptom-free volunteers aged 31–83 years were recruited by Fazekas, and MRI was performed for assessment. WML increased from 11% in the 4th decade to 83% in those over 70 years of age [21]. In our study, 79% of our subjects were found to have WML. Age was also found to be correlated with WML severity. Having a mean age of 71 years in our cohort, the prevalence and trend were comparable to the results by Fazekas.

Fazekas et al. introduced a grading system for WML assessment [15], which was a modification of the system proposed by Brant-Zawadzki et al. [22] and Zimmerman et al. [20]. The system involves the assessment of both periventricular hyperintensity and deep white matter hyperintensity. In our current study, we focused on deep white matter hyperintensity. The difference between periventricular and deep white matter lesions has yet to be well defined. Some studies have proposed that their clinical implication and pathological features are diverse. Barber et al. looked into MR images of patients with Alzheimer's disease and dementia with Lewy bodies [23]. Periventricular hyperintensities were found to independently correlate with atrophic processes involving ventricular enlargement. On the contrary, deep white matter hyperintensities did not correlate with measures of brain atrophy, but they were associated with ischaemic risk factors. Furthermore, histopathological studies have shown that deep white matter hyperintensity is related to the severity of ischaemic tissue damage [24]. As we postulated that lower urinary tract symptoms in the context of pelvic arterial insufficiency and ischaemia-related bladder degeneration were correlated to chronic ischaemic brain disease in the elderly, deep white matter hyperintensity on MRI was adopted for analysis in this study.

It has been proposed that WML is the pathoanatomic substrate in the brain aetiology of OAB [25]. However, overall data on the association between lower urinary tract dysfunction and WML are scarce and contradictory. A few studies have been attempted to assess the correlation between WML and lower urinary tract symptoms. In a smaller scale study by Sakakibara et al., they studied the urinary symptoms and WML of sixty-three subjects [26]. Among them, 33 subjects had urodynamic studies. WML was classified from grade 0 to grade 4 according to Brant-Zawadzki et al. [22]. In subjects with grade 1–4 WML, detrusor overactivity was more common (82%) than in those with grade 0 WML (9%). A similar result could not be demonstrated by our study probably due to quite a different composition of the cohort. In our study, most of the subjects had no or very mild WML. In contrast, the cohort of Sakakibara et al. had more subjects with more significant WML. In their study, 11 subjects belonged to grade 0, 15 subjects belonged to grade 1, 12 subjects belonged to grade 2, 14 subjects belonged to grade 3, and 11 subjects belonged to grade 4. Furthermore, the mean mini-mental state examination score was in the lower normal range in their cohort. These factors might signify that if there is a correlation between WML and LUTS, such a relationship would only be obvious if there were significant WMLs accompanied by some degree of neurological symptoms.

The association between urinary symptoms and WML was also investigated in the Leukoaraiosis And DISability (LADIS) study [27]. The LADIS study was a longitudinal, multicenter, observational study which primarily aimed at assessing the role of WML as an independent predictor of the transition to disability in initially nondisabled elderly persons. Six hundred thirty-nine subjects aged between 65 and 84 years were enrolled. Concerning urinary complaints, its assessment was done by 4 questions with the dichotomous answer yes or no. These questions were on nocturia, frequency, incontinence, and urgency. Except for urgency, no statistically significant difference was found between severity of WML and symptoms of nocturia, frequency, and incontinence. In fact, these few symptoms were found to be more prominent in those with mild WML than in those with moderate WML. Such contradictory findings could again be explained by the fact that the LADIS study had its cohort composition lying between our study and the study by Sakakibara et al. [26]. In our cohort, the majority of subjects (67%) with WML belonged to Fazekas grade 1. In comparison, the cohort of Sakakibara et al. had 29% of the subjects belonging to mild WML, and the cohort in the LADIS study had 44% of the subjects belonging to mild WML. Furthermore, the urological assessment in the LADIS study was not based on a validated questionnaire. Rather, it was based on 4 questions only. On the contrary, our study using a validated questionnaire of OAB-V8 offered a more proper assessment of the urological symptoms.

The results of our study were echoed by Wehrberger et al. [28]. Both the studies could not demonstrate a clear relationship between WML and LUTS. In the cohort of 217 patients by Wehrberger et al., urgency and nocturia were found to be more prevalent in subjects without WML. On the contrary, frequency was found to be more prevalent in subjects with WML. Furthermore, the WML burden did not correlate with LUTS. In general, data from both the studies failed to illustrate an obvious association between the presence of WML and LUTS. And unlike other studies, both the studies employed a standardized validated questionnaire for assessment, namely, Bristol LUTS questionnaire for Wehrberger et al. and OAB-V8 for our study.

Besides WML, there are a number of medical conditions that are associated with OAB. This includes bladder outlet obstruction. Absence of this information from our subjects for inclusion into analysis was one of the limitations of our study. However, as our cohort demonstrated only very mild symptoms of OAB, with an overall prevalence compatible with our age group, these concomitant medical conditions associated with OAB might not affect our analysis in the study.

WML is associated with age and is common in the elderly population. Mild WML is usually subclinical, with no obvious neurological and urinary symptoms. Our study on a cohort of 431 community-dwelling healthy elderly did not show any relationship between WML and LUTS. Further study would be needed to confirm the sequential symptoms manifestation with respect to an increasing severity of WML.

## Figures and Tables

**Table 1:** Questionnaire response characteristics.

Subjects in the cohort (*n*)	800
Respondents (*n*)	431
Male	159
Female	272
Median age, years (IQR)	70 (8)
Patients on anticholinergics (*n*)	9
Patients on beta-3 agonists (*n*)	0
Deep white matter Fazekas score	
0 (*n*)	91
1 (*n*)	227
2 (*n*)	89
3 (*n*)	24

IQR = interquartile range.

**Table 2:** Respondents' OAB-V8 results.

Respondents (*n*)	431		
OAB-V8	Nil	Mild	Moderate to severe
Urgency (Q2 + Q3 + Q7) (*n*)	290	114	27
Frequency (Q1) (*n*)	322	59	50
Nocturia (Q5 + Q6) (*n*)	213	137	81
Incontinence (Q4 + Q8) (*n*)	297	115	19

OAB-V8 = Overactive Bladder-Validated 8-Question Questionnaire. Question scores and classification: urgency: nil (0), mild (1–6), and moderate to severe (7–15); frequency: nil (0), mild (1-2), and moderate to severe (3–5); nocturia: nil (0), mild (1–4), and moderate to severe (5–10); incontinence: nil (0), mild (1–4), and moderate to severe (5–10).

**Table 3:** Generalized linear model of factors affecting lower urinary tract symptoms.

	OAB-V8 total score			OAB-V8 urgency score			OAB-V8 frequency score		
	Coefficient	SE	*p*	Coefficient	SE	*p*	Coefficient	SE	*p*
Age	−0.012	0.013	0.379	<0.001	0.020	0.982	−0.072	0.026	0.005
Male	0.243	0.153	0.112	−0.323	0.282	0.253	0.373	0.252	0.139
Fazeka score >0	−0.126	0.189	0.504	−0.202	0.293	0.491	0.309	0.306	0.314
Hypertension	0.081	0.154	0.600	−0.111	0.232	0.632	0.194	0.241	0.422
Diabetes mellitus	−0.082	0.158	0.604	−0.110	0.279	0.694	−0.077	0.259	0.765
Hyperlipidaemia	−0.024	0.151	0.875	−0.192	0.239	0.421	−0.155	0.250	0.534
Alpha blocker	0.287	0.194	0.139	0.097	0.432	0.822	0.932	0.322	0.004

OAB-V8 = Overactive Bladder-Validated 8-Question Questionnaire; SE = standard error; *p *= *p* value.

**Table 4:** Multiple logistic regression analysis of factors predicting OAB (OAB-V8 score >8).

Predictor	Adjusted OR	95% CI	*p* value
Age	0.99	(0.94, 1.04)	0.632
Male	1.29	(0.80, 2.09)	0.296
Fazeka score >0	1.13	(0.65, 1.96)	0.659
Hypertension	1.22	(0.74, 1.99)	0.433
Diabetes mellitus	0.94	(0.56, 1.58)	0.827
Hyperlipidaemia	1.19	(0.73, 1.93)	0.495
Alpha blocker	1.55	(0.73, 3.27)	0.253

OAB-V8 = Overactive Bladder-Validated 8-Question Questionnaire.

## Data Availability

The data of this study are not openly available to the public.
